# Successful Defibrillation After Conversion From Fixed‐Tilt to Tuned‐Duration Waveform

**DOI:** 10.1002/joa3.70436

**Published:** 2026-07-24

**Authors:** Yousaku Okubo, Hisayasu Matsuzaki, Shogo Miyamoto, Hiroshi Oe, Yukiko Nakano

**Affiliations:** ^1^ Department of Cardiovascular Medicine Hiroshima University Graduate School of Biomedical and Health Sciences Hiroshima Japan; ^2^ Division of Clinical Engineering, Clinical Support Department Hiroshima University Hospital Hiroshima Japan

**Keywords:** DeFT response, high defibrillation threshold, implantable cardioverter‐defibrillators, sudden cardiac death, tuned defibrillation waveforms, ventricular fibrillation, ventricular tachycardia

## Abstract

Fixed‐tilt biphasic shocks may become ineffective in high‐impedance states because phase‐2 over‐prolongation disrupts optimal charge neutralization. Waveform tuning based on membrane time constants, as implemented by DeFT Response, shortens phase durations to physiologic ranges and lowers the defibrillation threshold. This mechanism‐based strategy may enhance the reliability of ICD therapy in patients with rising impedance or high DFTs.

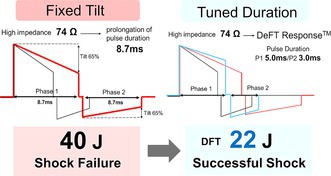

High defibrillation threshold (DFT) remains a clinically relevant problem in a subset of patients with implantable cardioverter‐defibrillators (ICDs) [1]. Although modern devices can deliver 35–40 J, DFT may increase over time owing to disease progression, antiarrhythmic drugs, or changes in lead characteristics, occasionally resulting in defibrillation failure. Most ICDs employ fixed‐tilt biphasic waveforms, in which phase duration automatically prolongs when high‐voltage lead impedance is high. Excessively prolonged phase‐2 duration is undesirable, as it may impair defibrillation efficacy by generating residual myocardial charge rather than neutralizing the phase‐1 voltage gradient. Abbott's DeFT Response algorithm allows individualized programming of pulse durations based on estimated cardiac membrane time constant and measured high‐voltage lead impedance, and several reports have demonstrated reduced DFTs with tuned‐duration waveforms [2, 3, 4]. Here, we describe a case of recurrent ventricular tachycardia in which fixed‐tilt shocks failed, but conversion to a tuned‐duration waveform enabled successful defibrillation with an adequate safety margin.

A 61‐year‐old man with idiopathic ventricular tachycardia (VT) underwent implantation of a single‐coil Durata defibrillation lead and a Gallant ICD (Abbott) 3 years earlier for secondary prevention. At implantation, the high‐voltage lead impedance in the RV–can configuration was normal (56 Ω), and all lead parameters were within normal limits. The device was programmed with a VT zone at ≥ 187 bpm, delivering three sequences of antitachycardia pacing (ATP) followed by shocks at 25, 36, 40, and 40 J, and a VF zone at ≥ 230 bpm with six shocks at 40 J. One year after implantation, an appropriate 25‐J shock successfully terminated sustained VT. At that time, the high‐voltage lead impedance in the RV–can configuration was 56 Ω, and the pulse durations were 5.8 ms for both Phase 1 and Phase 2. Subsequently, the patient was treated with multiple antiarrhythmic medications, including amiodarone 100 mg/day, carvedilol 20 mg/day, and mexiletine 300 mg/day. Two years later, while climbing stairs, he experienced palpitations and near‐syncope. Device interrogation revealed multiple ATP sequences followed by four shocks (25, 36, 40, and 40 J), none of which terminated monomorphic VT (Figure 1). He was transported to the hospital for further evaluation.

**FIGURE 1:**
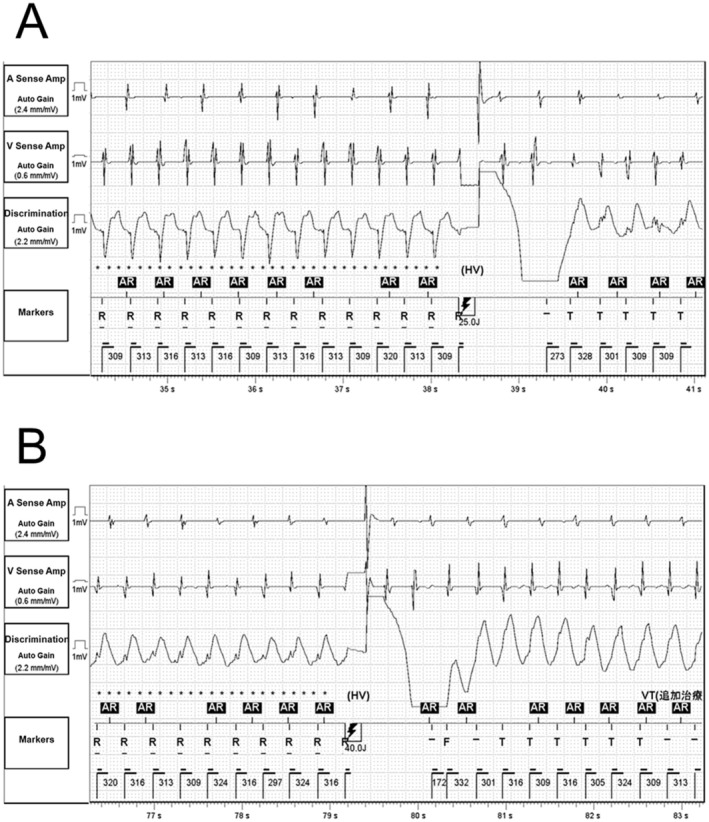
Intracardiac electrograms during unsuccessful defibrillation attempts. (A) Sustained monomorphic VT persisted despite multiple antitachycardia pacing (ATP) attempts and a 25‐J shock. (B) Additional 36‐J and 40‐J shocks also failed to terminate the arrhythmia, despite maximum ICD output.

On admission, ICD interrogation revealed a marked increase in high‐voltage lead impedance to 74 Ω. With the fixed‐tilt biphasic waveform (65%/65%), the high impedance resulted in automatic prolongation of pulse durations to 8.7 ms in both phases.

A defibrillation threshold (DFT) test was subsequently performed. Using the DeFT Response algorithm, the ventricular membrane time constant was estimated by measuring the peak‐to‐peak interval of the evoked potential during pacing (120 bpm × 8 beats). (Figure 2A) The measured value (74 ms) corresponded to the “Block 1” category. Combined with the high‐voltage lead impedance of 74 Ω, the calculated optimal pulse durations were 5.0 ms for Phase 1 and 3.0 ms for Phase 2. (Figure 2B) With these tuned‐duration settings, repeat DFT testing successfully terminated ventricular fibrillation with the first 22‐J shock, achieving a safety margin exceeding 10 J (Figure 3).

**FIGURE 2:**
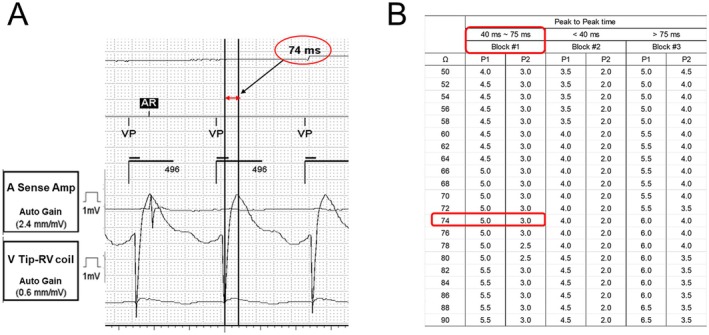
Estimation of membrane time constant and selection of tuned‐duration waveform. (A) Ventricular evoked response during pacing (120 bpm, eight beats) showed a peak‐to‐peak interval of 74 ms. (B) Based on the membrane time‐constant block and measured impedance (74 Ω), optimal durations were calculated as 5.0 ms (Phase 1) and 3.0 ms (Phase 2). (Table used with permission from Abbott.)

**FIGURE 3:**
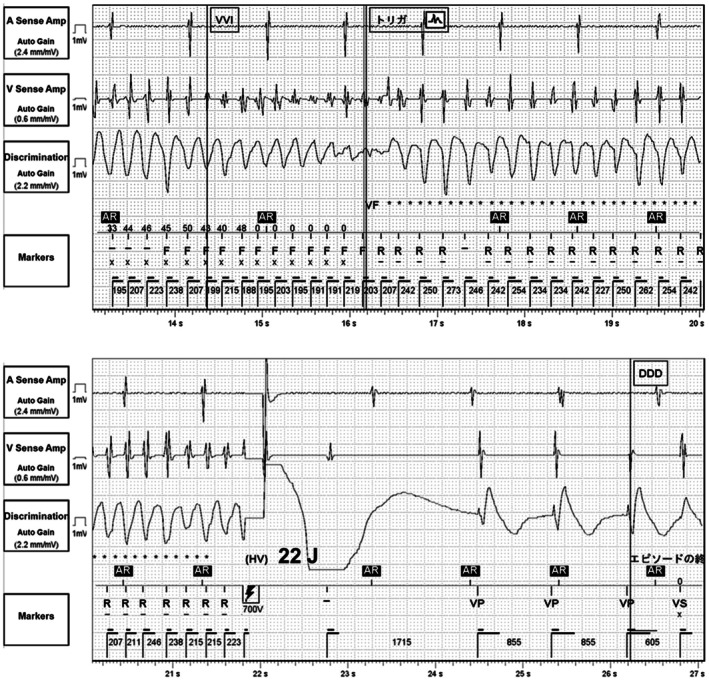
Successful defibrillation using a tuned‐duration waveform. During DFT testing with individualized phase durations (5.0/3.0 ms), a 22‐J shock successfully terminated ventricular fibrillation, restoring an adequate safety margin.

This case illustrates a clinically important situation in which the defibrillation threshold increased several years after ICD implantation despite previously normal lead parameters and an adequate safety margin. Chronic amiodarone therapy and a substantial rise in high‐voltage lead impedance—from 56 to 74 Ω—likely contributed to repeated shock failure. In this case, the routinely measured high‐voltage lead impedance in the RV–can configuration had already increased to 71 Ω before the second unsuccessful shock. In retrospect, earlier adjustment of the waveform from fixed tilt to tuned pulse durations might have prevented repeated defibrillation failure. Because fixed‐tilt waveforms prolong pulse duration as impedance increases, the delivered pulse width reached 8.7 ms in both phases, producing a waveform configuration that was physiologically unfavorable for defibrillation.

The mechanism underlying this failure can be partly explained by the charge‐burping concept [5, 6]. Phase‐1 of a biphasic shock reduces excitable myocardium by creating a strong transmembrane voltage gradient, whereas Phase‐2 is intended to neutralize the residual charge produced during Phase‐1. (Figure 4A) When Phase‐2 is delivered with an appropriate duration, the membrane potential returns toward baseline and post‐shock activation is suppressed. However, if Phase‐2 becomes excessively prolonged—as observed in this patient under high‐impedance conditions—the reversal of membrane charge may be exaggerated. (Figure 4B) Instead of neutralizing residual charge, an overly long Phase‐2 can generate reverse membrane charging, increasing the probability of early refibrillation even at maximum ICD output. Abbott's DeFT Response algorithm provides a mechanism‐based solution by calculating individualized phase durations using the patient's evoked response and measured high‐voltage lead impedance. Although excessive prolongation of phase duration under high‐impedance conditions may theoretically occur in any ICD system using fixed‐tilt biphasic waveforms, programmable pulse‐duration adjustment is currently available only in Abbott devices through the DeFT Response algorithm. In this case, the estimated membrane time constant and elevated impedance produced tuned durations of 5.0 ms for Phase‐1 and 3.0 ms for Phase‐2, substantially shorter than those generated by fixed tilt. After reprogramming, defibrillation was successful at 22 J, restoring a safe margin without invasive system modification. Clinical studies have demonstrated that appropriately timed millisecond‐duration waveforms reduce DFT, particularly in patients with high impedance [7]. The present case reinforces this concept by showing that tailored pulse durations can overcome impedance‐related waveform distortion and restore effective defibrillation. Tuned‐duration programming represents a simple, noninvasive, and mechanistically rational strategy for managing elevated DFT, especially in patients with rising impedance, single‐coil systems, or chronic amiodarone exposure.

**FIGURE 4:**
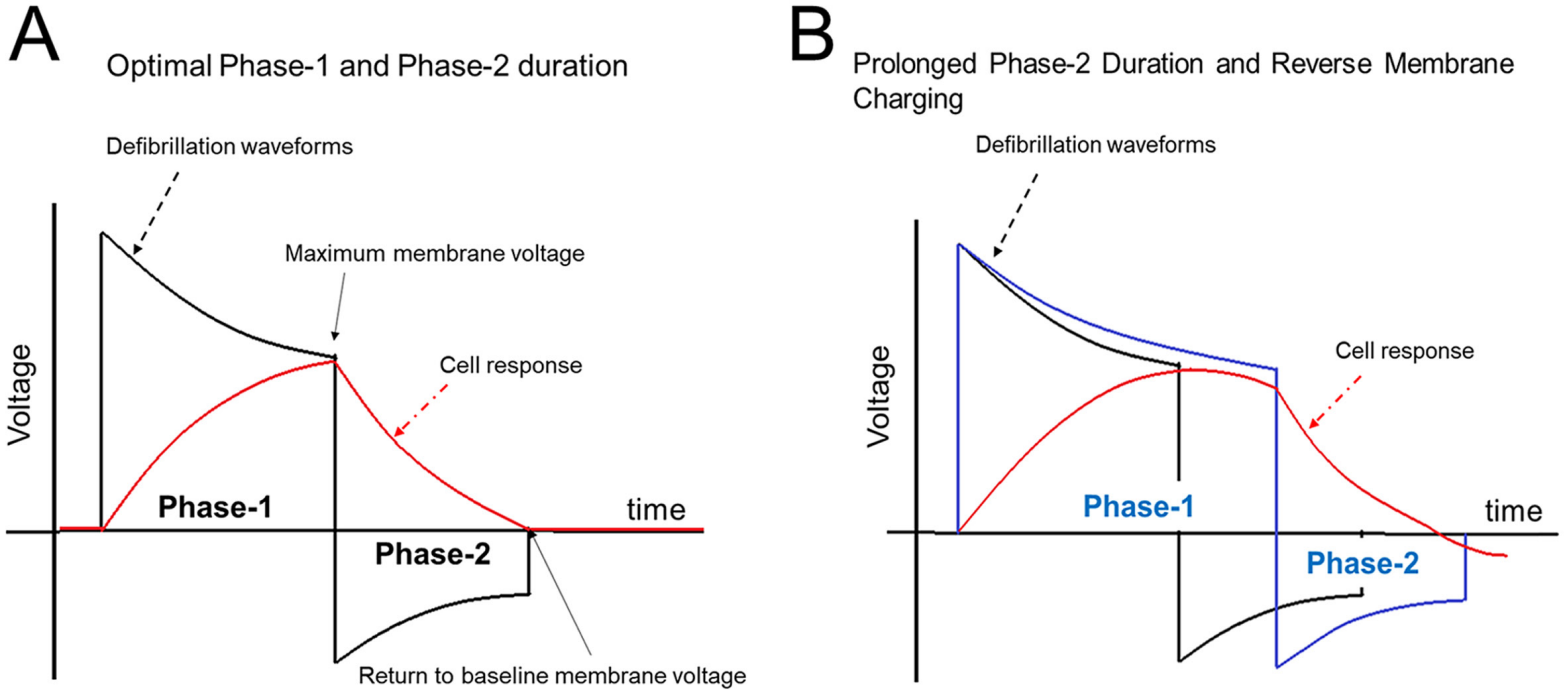
Defibrillation waveforms and membrane response curves. (A) Appropriately timed Phase‐1 and Phase‐2 pulses produce effective depolarization and charge neutralization (“charge‐burping”), suppressing post‐shock activation. (B) Excessive Phase‐2 prolongation, as seen under high‐impedance conditions, causes reverse membrane charging and increases the likelihood of refibrillation.

## Funding

The authors did not receive any specific funding for this study.

## Ethics Statement

Approval was obtained from the local ethics committee.

## Consent

The authors obtained consent from the patients.

## Conflicts of Interest

The authors declare no conflicts of interest.

## Data Availability

The data that support the findings of this study are available on request from the corresponding author. The data are not publicly available due to privacy or ethical restrictions.
